# Application of Induced Pluripotent Stem Cell-Derived Models for Investigating microRNA Regulation in Developmental Processes

**DOI:** 10.3389/fgene.2022.899831

**Published:** 2022-05-26

**Authors:** Hongyu Chen, Mimi Zhang, Jingzhi Zhang, Yapei Chen, Yabo Zuo, Zhishen Xie, Guanqing Zhou, Shehong Chen, Yaoyong Chen

**Affiliations:** ^1^ Department of Obstetrics and Gynecology, Key Laboratory for Major Obstetric Diseases of Guangdong Province, The Third Affiliated Hospital of Guangzhou Medical University, Guangzhou, China; ^2^ Key Laboratory of Reproduction and Genetics of Guangdong Higher Education Institutes, The Third Affiliated Hospital of Guangzhou Medical University, Guangzhou, China; ^3^ Guangzhou Key Laboratory for Clinical Rapid Diagnosis and Early Warning of Infectious Diseases, KingMed School of Laboratory Medicine, Guangzhou Medical University, Guangzhou, China

**Keywords:** microRNA, induced pluripotent stem cell, cellular model, develoment, gene regulaiton

## Abstract

Advances in induced pluripotent stem cell (iPSC) techniques have opened up new perspectives in research on developmental biology. Compared with other sources of human cellular models, iPSCs present a great advantage in hosting the unique genotype background of donors without ethical concerns. A wide spectrum of cellular and organoid models can be generated from iPSCs under appropriate *in vitro* conditions. The pluripotency of iPSCs is orchestrated by external signalling and regulated at the epigenetic, transcriptional and posttranscriptional levels. Recent decades have witnessed the progress of studying tissue-specific expressions and functions of microRNAs (miRNAs) using iPSC-derived models. MiRNAs are a class of short non-coding RNAs with regulatory functions in various biological processes during development, including cell migration, proliferation and apoptosis. MiRNAs are key modulators of gene expression and promising candidates for biomarker in development; hence, research on the regulation of human development by miRNAs is expanding. In this review, we summarize the current progress in the application of iPSC-derived models to studies of the regulatory roles of miRNAs in developmental processes.

## Introduction

MiRNAs are short RNA molecules with 20–24 nucleotides that regulate the posttranscriptional silencing of target genes (Krol et al., 2010; Fabian and Sonenberg, 2012; Luo and Zhu, 2014; Lu and Rothenberg, 2018). MiRNAs exhibit a complex regulatory network resulting from a particular miRNA targeting multiple mRNAs and multiple miRNAs targeting the same mRNA, and affecting the expression levels of many protein-coding genes involved in functional pathways (Liu et al., 2014; Barwari et al., 2016; Luo et al., 2016; Rupaimoole and Slack, 2017). Over the past few decades, the role of miRNAs has been evaluated in a variety of biological processes (Ambros, 2004; Luo et al., 2015b; Lopez et al., 2017; Song et al., 2019). To date, numerous studies have delineated the regulatory role of miRNAs in development. For instance, miRNAs are regulating cell differentiation, proliferation, apoptosis and migration during B cell development by regulating a spectrum of signalling pathways, including BCR, MAPK/ERK, PI3K/AKT and NFκB pathways (Katsaraki et al., 2021). Thus, miRNAs have been characterized as valuable modulators of human development.

The investigation of miRNAs in development requires *in vitro* models derived from human pluripotent stem cells to simulate the tissue developmental procedures. Nevertheless, there are a number of shortages of human embryonic stem cell (hESC) techniques, such as ethical issues and complicated manipulation, thus preventing its wide application in clinical and basic research (Barker and de Beaufort, 2013; Luo et al., 2014). In 2006, studies were conducted to reprogram somatic cells into pluripotent stem cells with a cocktail of transcriptional factors, such as the combination of OCT4, KLF4, SOX2 and c-Myc (Takahashi and Yamanaka, 2006). This method avoids moral controversies and has led to the application of cellular programming techniques in human developmental research (Lo Sardo et al., 2017). Thus, the emergence of human induced pluripotent stem cells (hiPSC) has solved these problems (Luo et al., 2015a).

**FIGURE 1:**
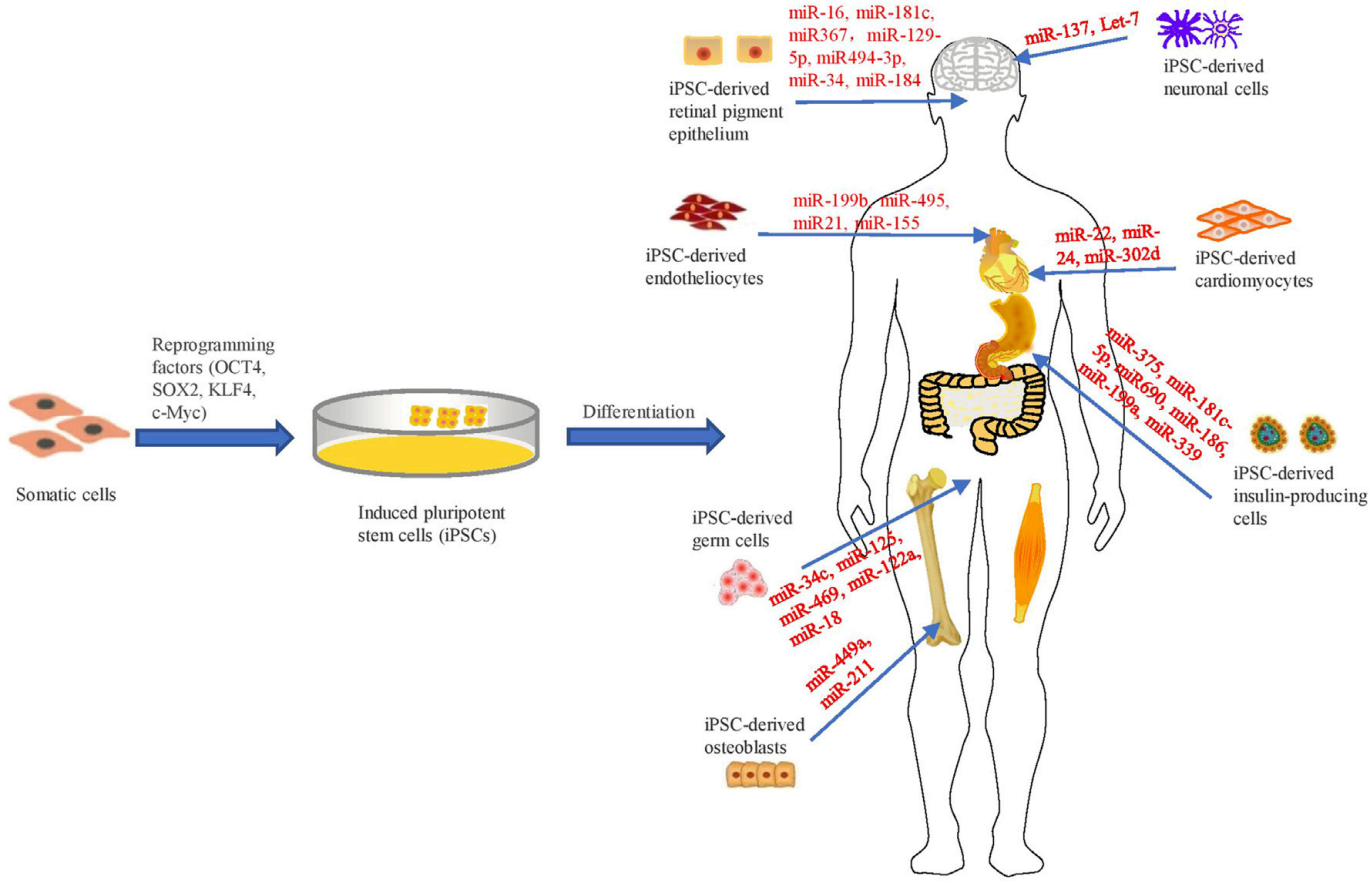
Schematic representation of investigating the regulatory roles of miRNAs in iPSC-derived cellular models.

Remarkable progress has been created within the area of hiPSC over the past decade (Luo et al., 2018; Luo et al., 2021a). At present, hiPSCs can specifically differentiate into cardiomyocytes, endothelial cells, insulin-producing cells, germ cells, neuronal cells, osteoblasts, retinal pigment epithelium and so on (Figure 1). These cells could be ultilized for research of human development and diseases (Luo et al., 2021c). Hence, this review aims to systematically summarize the regulatory roles of miRNAs in development identified by iPSC-derived models.

## Cardiomyocytes

Cardiovascular diseases (CVDs), such as myocardial infarction (MI) and cardiomyopathy, are recognized as the leading lethal causes around the world and are often associated with degeneration of cardiomyocytes (CMs). CMs are fully differentiated cells with minimal proliferative potential. Given the restricted effectiveness of drug therapy in treating myocardial injuries, the development of novel therapeutic approaches for curing these disorders is of urgency. HiPSC-derived CMs (iPS-CMs) introduce a new prospect for CVD treatment. However, the molecular mechanisms regulating the development of these cells is a pivotal problem that should be solved prior to clinical usage.

For instance, a study has compared the mRNA and miRNA expression profiles of iPS-CMs and biopsies from fetal, adult and hypertensive hearts to find out the core miRNA network, which revealed miRNAs associated with human heart development (Babiarz et al., 2012). Further studies profiled the miRNAs in human iPS-CMs and revealed 96 miRNAs that could promote CM proliferation (Diez-Cuñado et al., 2018). The CM proliferation-associated miRNAs in human were quite different from those of rodent (Eulalio et al., 2012). Most human CM proliferation-associated miRNAs function by targeting the Hippo pathway, an evolutionarily conserved pathway regulating organ size (Yu and Guan, 2013). Another study also confirmed that the mRNAs encoding most components of the Hippo pathway were recruited into the RNA-induced silencing complex (RISC) in iPS-CMs (Diez-Cuñado et al., 2018). In addition, some studies have demonstrated that miR-302d promoted CM proliferation by inhibiting LATS2 of the Hippo pathway (Xu F. et al., 2019).

Recently, miR-24 has been demonstrated as an important regulator for human heart development by using iPS-CM models (Guo et al., 2015). This is an execllent example of the complex regulatory roles of miRNAs in human development. On one hand, miR-24 has been demonstrated to suppress CM apoptosis. It is shown that delivery of miR-24 into CMs significantly alleviates cardiomyopathies, suggesting that modulating miRNA levels might be a novel therapeutic means for cardiac diseases (Qian et al., 2011; Guo et al., 2015). One study showed that miR-24 promoted functional implantation of cardiovascular progenitor cells (CPCs), in which miR-24 was utilized as a component of the antiapoptotic cocktail to enhance the survival of CPCs implantated into the MI heart tissues (Hu et al., 2011). Other studies have also identified other prosurvival roles of miR-24 in cardiac fibrosis and found that overexpression of miR-24 through lentivirus-mediated transduction reduces fibrosis and improves cardiac function in MI hearts, confirming the beneficial role of miR-24 (Guo et al., 2015). On the other hand, miR-24 has been demonstarted to exert proapoptotic effects. MiR-24 is characterized as a proapoptotic miRNA in cardiac endothelial cells, and blocking its function by injection of miR-24 antagonists can prevent apoptosis, enhance vascular distribution, and improve cardiac function after MI (Fiedler et al., 2011). However, these experiments in earlier studies were performed by viral transduction or polymeric transfection of miR-24 mimics or inhibitors, in which the protective effect observed might be partly caused by their off-target effects in non-CM cells (Guo et al., 2015).

In addition, some studies have also found that pri-miR-22/miR-22-3p is the top-ranked expressed primary miRNA transcript in heart tissues and iPS-CMs, and contributes to myocardial ischemia/reperfusion injury (Du et al., 2016; Sun et al., 2019). Some studies have confirmed the pro-apoptotic effect of miR-22 in iPS-CM (Pan and Zhu, 2018; Sun et al., 2019). Hypoxia-mediated apoptosis was augmented by miR-22 overexpression but resuced by miR-22 knockdown in iPS-CMs (Gidlöf et al., 2020). Meanwhile, this study also demonstrated that the long non-coding RNA Neat1 in the paraspeckles is the essential factor for pri-miR-22 processing in CMs. Knockdown of Neat1 could lead to significant accumulation of pri-miR-22 and consumption of mature ones in iPS-CMs (Gidlöf et al., 2020).

## Vascular Endothelial Cells

MiR-199b is a highly conserved miRNA across species and capable of guiding the hiPSCs to differentiate into vascular endothelial cells (ECs) by regulating key molecular pathways, such as Notch signaling, in response to angiogenic signals. In particular, miR-199b regulates EC fate by targeting the Notch ligand JAG1, which leads to expression and secretion of VEGF via STAT3-mediated trascription (Chen et al., 2015). Nevertheless, the molecular mechanism underlying the upstream regulation remains unclear. Moreover, VEGF-induced miR-155 promotes angiogenesis by directly silencing E2F2, a E2F family transcriptional factor involved in cell proliferation, apoptosis and death (Dimova and Dyson, 2005), during EC differentiation from hiPSCs (Yang et al., 2016).

MiR-495 is a member of the DLK1-Dio3 miRNA cluster and exerts antiangiogenic effects. It is abundant in the non-EC portion while downregulated in the EC portion. It induces endothelial or angiogenic gene expression by downregulating VEZF1, a major transcriptional factor regulating EC genes, such as IGF1 and CD31, during EC differentiation and angiogenesis (Zou et al., 2010). In contrast, increasing VEZF1 expression via miR-495 blockage promotes angiogenesis post implantation of hiPSCs via enhancement of EC production. Studies have shown that the derived ECs significantly augmented the formation of new blood vessels in infarcted hearts, prevented functional deterioration and restricted the expansion of infarcted areas post transplantation in MI mice (Liang et al., 2017).

Additionally, miR-21 overexpression could enhance the Akt/TGF-β2 signal by downregulating PTEN on chromosome 10, thereby increasing the amount of ECs derived from hiPSCs (Zeng et al., 2018). Overexpression of miR-21 increased the mRNA and protein levels of TGF-β2, which is an essential cytokine for cell survival proliferation, migration and differentiation (Vargel et al., 2016). Neutralizing TGF-β2 by antibodies prohibits the expression of miR-21-induced EC markers, such as VE-CAD and CD31 (Di Bernardini et al., 2014).

## Insulin-Producing Cells

The generation of insulin-producing cells (IPCs) from hiPSCs is a promising approach to investigate the molecular mechanisms of pancreatic development and a potential source of treatment for type I diabetes (Zeng et al., 2018). MiRNAs are major posttranscriptional regulators of gene expression and thus might involve in the control of β cell development in the pancreas.

For examples, miR-375 is essential for pancreatic endocrine function as its blockage results in glucose imbalance, α cell increment and β cell reduction (Poy et al., 2009). MiR-375 and miR-186 overexpression in hiPSCs leads to differentiation into insulin-secreting β-like cells that expressing pancreatic endocrine markers, such as PDX1, GLUT2, NGN3, PAX4 and PAX6. Despite secreting less insulin than natural β cells, these hiPSC-derived β-like cells could rescue blood glucose levels after transplantation into diabetic mice (Shaer et al., 2014). In addition, miR-375 affects insulin secretion by regulating the expression of muscular dystrophy protein in MIN6 cells (Poy et al., 2004; Krek et al., 2005).

The development of organisms is a result of the reprogramming of gene regulatory networks (Hornstein and Shomron, 2006). Some studies have described miR-375 as a key regulator of pancreatic development in humans (Poy et al., 2004; Lynn et al., 2007; Bravo-Egana et al., 2008; Avnit-Sagi et al., 2012). Mice lacking miR-375 showed α/β cell imbalance and reduced β cell propagation in spite of insulin insufficiency (Avnit-Sagi et al., 2012). Studies have shown that miR-7, miR-9, miR-375 and miR-376 are dramatically upregulated throughout the islet development (Wei et al., 2013). Some studies have found that miR-186, miR-199a and miR-339 are also upregulated during the formation of IPCs *in vitro*. The target genes of these three microRNAs include LIN28, PRDM1, CALB1, GCNB2, RBM47, PLEKHH1, RBPMS2 and PAK6 mRNA. (Joglekar et al., 2009; Chen et al., 2011; Kredo-Russo et al., 2012).

Studies have shown that miR-181c-5p accumulates gradually during the derivation of IPCs from hiPSCs. Increased phosphorylation of Smad2/3 is observed in iPSC-derived cells, and treatment with a Smad2/3 inhibitor after overexpression of miR-181c-5p had the opposite effect on IPC formation (Li et al., 2020). Similarily, other studies have also shown that miR-181c-5p is abundant in the late differentiation steps of hESC-derived IPCs, fetal pancreas, and adult islets (Liao et al., 2013; Fogel et al., 2015). Furthermore, miR-181c-5p was differentially expressed between the pancreas and the liver despite the common developmental origin of both tissues, with upregulation in the former and downregulation in latter (Porciuncula et al., 2013). Therefore, it is speculated that miR-181c-5p might play a pancreatic-specific role.

On the other hand, miR-690 overexpression dramatically delayed iPSC-derived IPC maturation and reduced insulin secretion *in vitro* and *in vivo*. Bioinformatic analysis suggested that its putative targets, such as CTNNB1, STAT3 and SOX9, were essential factors for of pancreatic endocrine development. Elevated miR-690 expression levels disrupt IPC differentiation by directly binding to Sox9. Subsequent experimental studies suggest that miR-690 could negatively modulate the Wnt signalling pathway during the pancreatic developmental process (Xu Y. et al., 2019).

In conclusion, these findings may help us better understand the process of pancreatic differentiation of hiPSCs *in vitro* and the underlying mechanisms involving miRNAs. As miRNAs could modulate certain transcriptional factors throughout the pancreatic developmental process, they could serve as novel therapeutic targets for diabetes treatment.

## Neuronal Cells

HiPSC-derived neurons and neural progenitor cells (NPCs) are important models for investigating neurogenesis and synaptogenesis as well as their disruption in disorder statuses. Moreover, they are promising therapeutic vectors for brain disorders in the future (Zhu et al., 2013; Zhu et al., 2014). HiPSC-derived cellular and organoid models serve as an important bridge between model organism research and human postmortem brain research by providing living human cells, consisting of hiPSCs and their derived NPCs and neurons, with the composite genetic background present in patients. Hence, there is a quickly growing body of research projects using patient-specific iPSC-derived neurons to investigate neurogenesis.

For instance, iPSCs with mutations in the LRRK2 and α synuclein gene families were used to generate dopamine (DA) neurons, which exhibited higher sensitivity to oxidative stress and susceptibility to apoptosis (Byers et al., 2011; Reinhardt et al., 2013). Such phenotypes were also observed in iPSC-derived DA neurons from idiopathic Parkinson’s disease (PD) patients (Sánchez-Danés et al., 2012); meanwhile, apoptotic markers were also detected in the postmortem brain of PD patients (Hartmann et al., 2000; Mogi et al., 2000). Apoptosis-related miRNAs are also associated with neuronal differentiation (Aranha et al., 2011). For examples, miR-14, let-7a and miR-34a are elevated during neural stem cell differentiation (Heman-Ackah et al., 2013).

A large number of investigations have demonstrated that miRNAs play important roles in neural development (Hsu et al., 2012; Jimenez-Mateos et al., 2012; Liu et al., 2012). In addition, abundant molecular evidences support the essential roles of miRNAs in development of schizophrenia and other neural diseases (Green et al., 2013). For instance, miRNA-seq analysis was performed to distinguish differentially expressed miRNAs in iPSC-derived neurons from schizophrenia patients with 22q11.2 deletions compared to those from healthy donors (Yang et al., 2010). They discovered that miRNA expression levels in the deleted region decreased to approximately half the normal levels, and the levels were also altered in several other miRNAs out of the deleted region. The functional annotations of the putative targets of these dysregulated miRNAs were enrich in neurological diseases, neuronal development, axon formation and other important pathways relevant with the nervous system (Zhao et al., 2015).

Finally, posttranscriptional modifications could be identified by transcriptome analysis. RNA editing is a posttranscriptional event. Adenosine to inosine (A-to-I) transition is the dominate RNA editing process and happens most frequently in RNA molecules relevant with neurotransmission (Sanjana et al., 2012), especially in a lot of brain-specific miRNAs (Nishikura, 2010). Intriguingly, comparing postmortem cerebellum of autism patients with the control ons discovered that RNA editing was more abundant in the autism samples (Eran et al., 2013). Total transcriptome analysis can also detect fusion genes (Zhang Y. et al., 2014), which is valuable for building coexpression networks that can help researchers discover gene networks and pathways that are disrupted in neuropsychiatric disorders.

MiRNAs are believed to exert key regulatory effects in a wide spectrum of neural developmental processes, such as neurogenesis, neuronal maturation, axon regeneration, synaptic development and brain plasticity (Giraldez et al., 2005; Weston et al., 2006). Let-7 family miRNAs are the highest expressed miRNAs in the mammalian brains (Lagos-Quintana et al., 2002). They were firstly identified in *Caenorhabditis elegans* (Roush and Slack, 2008) and highly conserved across species. They are the key regulators for organism development, such as cell proliferation, cell specification and terminal differentiation (Nishikura, 2010). In the developing brain, Let-7 miRNAs participate in control of various developmental processes, such as neuronal differentiation (Schwamborn et al., 2009), neuronal subtype specification (Weick et al., 2013), neuronal regeneration (Li et al., 2015) and synaptic formation (Edbauer et al., 2010). Albeit in silico models suggest that the Let-7 miRNAs are involved in modulating postsynaptic gene expression (Paschou et al., 2012), their direct functions in mature human neurons remain unclear.

One of the Let-7 members, Let-7c is located on chromosome 21; thus, it exists in an extra copy of trisomy 21 (T21) and is associated with the symptoms of mild to moderate mental retardatoin featured in this neurodevelopmental syndrome (Antonarakis, 2017). It has been reported that miRNAs encoded by chromosome 21 may be important for a comprehensive understanding of the pathophysiology of T21-related neural diseases (Izzo et al., 2017). Taken together, these investigations indicate that the Let-7 family plays an important role in modulating human neurodevelopment and provide clues to illustrating the complicated molecular aetiology of neurodevelopmental syndromes (McGowan et al., 2018).

## Germ Cells

In humans, genetic information is passed on to their offspring via germ cells (Luo et al., 2021b; Zhou et al., 2022). At present, ESCs, iPSCs and spermatogonial stem cells are the major cell sources used for generation of male germ cells expressing functional genes (Saito et al., 2015). However, their clinical utility is still challenged by several safety issues (Zhang D. et al., 2014). MiRNAs have recently emerged as important factors in translation regulation and the epigenetic control of stem cell self-renewal and pluripotent capacities (Gangaraju and Lin, 2009). Key roles of miRNA pathways in germline stem cell maintenance have been reported in vertebrate iPSCs (Gangaraju and Lin, 2009). In addition, miRNAs are very important in spermatogenesis and might play key roles in sperm mitosis, meiosis and postmeiotic stages (Wang and Xu, 2015).

The role of miRNAs in germ cell development has been functionally proven (Fernández-Pérez et al., 2018). For examples, RNA binding protein Lin28 blocks Let-7 and desuppresses Blimp1 translation in the initial stage of germ cell development (West et al., 2009). In addition, miR-125 posttranscriptionally suppress Oct4 during sperm meiosis in males (Medrano et al., 2013).

Further experimental evidence should be pursued to identify specific microRNAs that are regulating the three stages of human spermatogenesis, pachytene spermatocytes, spermatogonial cells and round spermatozoa cells (Liu Y. et al., 2015). For examples, miR-34c increased in pachytene spermatocytes and round sperm cells and prohibited survival by targeting the transcription factor ATF1 (Romero et al., 2011). In addition, miR-469 inhibited protamine and transition protein 2 (TP2) mRNA in pachytene spermatocytes and round sperm cells (Dai et al., 2011). Moreover, during spermatogenesis, miR-122a and miR-18 downregulate TP2 and heat shock factor 2, respectively (Chen et al., 2017).

## Retinal Pigment Epithelium

The retinal pigment epithelium (RPE) is a special layer arranged at the rear of retina. Injury or RPE dysfunction can severely affect the health of photoreceptors and visual function, which is a result of potential RPE pathological blinding disease. Examples include age-related macular degeneration (AMD), Stargardt disease and retinitis pigmentosa (Greene et al., 2014). So far, there is no efficient therapy to rescue the vision; thus, iPSC-derived RPE (iPS-RPE) cells might be a source of cells to regenerate the disrupted RPE. However, before iPS-RPE cells can be used clinically, as much information as possible about the factors that modulate RPE development is of urgency to increase the production and quality of the cells for therapeutic use (Greene et al., 2014).

A study has identified 155 potential miRNA markers in iPS-RPE cells (Wang et al., 2014). Upregulated miRNAs, such as miR-181c and miR-129-5p, might drive cell specification (Naguibneva et al., 2006; Ryan et al., 2006), while downregulated miRNAs, including miR-367, miR-18b and miR-20b, are associated with mitotic division (Budde et al., 2010; Murakami et al., 2013). Putative targets of these miRNAs are relevant with cell survival, cell cycle and development.

It is of interest to evaluate the possible role of iPS-RPE miRNAs in tumorigenesis. On one hand, some iPS-RPE-upregulated miRNAs are tumour suppressors. For instance, miR-34 is a typicall tumour suppressor that prohibits tumor growth, metastasis, invasion and epithelial-mesenchymal transformation (EMT) via downregulating TP53 (Zhang et al., 2007; Nana-Sinkam and Croce, 2013). MiR-34 is generally silenced in multiple cancer types. MiR-34 expression was amplified in iPS-RPE cells by 5-fold, indicating an extremely low proliferative capacity in these terminally differentiated cells (Hermeking, 2012). Similarly, miR-16 is a tumour suppressive miRNA targeting multiple oncogenes, including EGFR, JUN and BCL2. In contrast, many iPS-RPE-downregulated miRNAs are oncogenic miRNAs (Wang et al., 2014).

MiRNAs in extracellular vesicles (EVs) derived from RPE cells might exert effects in the malignant inflammatory cycle. A specific enrichment of miR-494-3p was identified in EVs secreted from iPS-RPE cells after interaction with MPs, which might be a potential therapeutic target for the treatment of AMD (Mukai et al., 2021). AMD is the first and the third top causes of blindness in developed countries and around the world, respectively (Kuo et al., 2012). MiR-184 on chromosome 15q25.1 is a highly conserved miRNA across species (Nomura et al., 2008). MiR-137 is gradually upregulated during the differentiation of hiPSCs into RPE cells and it will downregulate PKBβ (also known as Akt2), the major downstream effector of rapamycin (mTOR) signalling pathway (Jiang et al., 2016). Hence, dysregulation of miR-137 is an important molecular event during the progression of AMD (Jiang et al., 2016).

## Osteoblasts

HiPSCs could provide a rich cell source for regenerative medicine and to create patient-specific cellular and organoid models to investigate both intracellular and extracellular agents in bone repair and osteoarthritis (Diekman et al., 2012). Several histone deacetylase (HDAC) inhibitors have been shown to promote osteoblast maturation and specific gene expression by upregulating Runx2 gene expression in bone marrow stem cells (Hu et al., 2013). HDAC1 changes the expression of many genes associate with cell growth, survival, subtype specification and genome integrity (Buurman et al., 2012). One miRNA, miR-449a, specifically interferes with HDAC1 expression (Jeon et al., 2012; Okamoto et al., 2012). Exogenous miR-449a silencing endogenous HDAC1 expression keeps histone acetylation, induces Runx2 expression, which is a regulator of osteoblast genes (Nishimura et al., 2012), and accelerates osteoblast derivation from iPSCs (Liu T. et al., 2015).

In addition, an independent study demonstrated that a group of six miRNAs, miR-10a/b, miR-19b, miR-9, miR-124a, and miR-181a, are key regulators of the iPSC differentiation into osteoblasts (Okamoto et al., 2012). Moreover, another study has shown that miR-211 promoted iPSC differentiation into osteoblast-like cells via upregulating the expression of autophagy-related genes like ATG14 (Ozeki et al., 2017).

## Discussion

MiRNAs are functioning within the RNA-protein complexes known as RNA-induced silencing complexes (RISC), which regulates gene expression posttranscriptionally in higher eukaryotes (Ameres and Zamore, 2013). Their roles in human development are rapidly being discovered (Table 1). MiRNAs are undoubtedly involved in many stages of normal cell development through their ability to block or promote development. They can be regulated by epigenetics, which may lead to other regulatory effects. In addition, they could serve as valuable markers for patient diagnosis and prognosis, as well as promising therapeutic targets. Although the multifaceted role of miRNAs in some diseases has been extensively studied over the past few years, important information is still missing, and no single molecule has been proven to be an effective regulator of the many pathogenic pathways of disease (Katsaraki et al., 2021).

**TABLE 1:** Roles of miRNAs in development of various iPSC-derived cell lineages.

iPSC-Derived Cell Lineages	miRNA	Target	Effect	References
Cardiomyocytes	miR-24	Bim	Inhibit apoptosis	Guo et al. (2015)
	miR-22	HIF1A/SIRT1	Promote apoptosis	Du et al., 2016
				Sun et al., 2019
				Gidlöf et al. (2020)
	miR-302d	LATS2	Promote cell proliferation	Xu et al. (2019a)
Endotheliocyte	miR-199b	JAG1	Promote transcription, activation and secretion of VEGF	Chen et al., 2015
				Yang et al., 2016
				Dimova and Dyson, (2005)
	miR-495	VEZF1	Inhibit EC differentiation and angiogenesis	Zou et al., 2010; Liang et al., 2017
	miR-21	PTEN/VE-cad/CD31	Promote cell proliferation and differentiation	Zeng et al., 2018
				Di Bernardini et al., 2014
				Vargel et al. (2016)
	miR-155	E2F2	promotes angiogenesis	Dimova and Dyson, 2005
				Yang et al. (2016)
Insulin-producing cells	miR-375	HNF6/INSM1/PDX1	Its increase promotes islet formation and its decrease promotes ß -cell maturation and function	Shaer et al., 2014
				Poy et al., 2004
				Krek et al., 2005
				Hornstein and Shomron, 2006
				Lynn et al., 2007
				Bravo-Egana et al., 2008
				Avnit-Sagi et al., 2012
				Wei et al. (2013)
				(Joglekar et al., 2009
				Chen et al., 2011
				Kredo-Russo et al. (2012)
	miR-181c-5p	Smad7/TGIF2	Maintain cell-specific function	Li et al., 2020
				Liao et al., 2013
				Fogel et al. (2015)
	miR-690	SRY-Sox9	Inhibit cell differentiation and insulin production	Xu et al. (2019b)
	miR-186, miR-199a, miR-339	LIN28/PRDM1/CALB1/GCNB2/RBM47/PLEKHH1/RBPMS2/PAK6	Formation of IPCs *in vitro*	Joglekar et al., 2009
				Chen et al., 2011
				Kredo-Russo et al. (2012)
Neuronal cells	miR-137	NRXN1	Inhibit synaptic growth and maturation in the hippocampus and cortical	Green et al. (2013)
	Let-7	LIN28B	Regulates neuronal differentiation, neuronal subtype regulation and synaptic formation, as well as cell cycle regulation and tumor suppression	Giraldez et al., 2005
				Weston et al., 2006
				Lagos-Quintana et al., 2002
				Roush and Slack, 2008
				(Schwamborn et al., 2009
				Weick et al., 2013
				Li et al., 2015
				Edbauer et al., 2010
				Paschou et al., 2012
				Antonarakis, 2017
				Izzo et al. (2017)
Germ cells	miR-34c	ATF1	Cause round sperm cells and trigger apoptosis	Romero et al. (2011)
	miR-125	Oct4	Inhibit sperm meiosis	Medrano et al. (2013)
	miR-469	TP2	Inhibit sperm meiosis	Dai et al. (2011)
	miR-122a, miR-18	TP2/heat shock factor 2	Involved in spermatogenesis	Chen et al. (2017)
Retinal pigment epithelium	miR-16	BCL2/JUN/EGFR	Inhibits cell proliferation, epithelial-mesenchymal transformation (EMT), metastasis, and invasion and acts as a strong tumor suppressor	Zhang et al., 2007
				Nana-Sinkam and Croce, (2013)
	miR-181c	HOX-A11	Promote cell differentiation	Naguibneva et al., 2006
				Ryan et al. (2006)
	miR-129-5p	CDK6/EIF2C3/CAMTA1	Antiproliferative effect	Naguibneva et al., 2006
				Ryan et al. (2006)
	miR-367	HDAC2	Downregulation is associated with cell proliferation	Budde et al., 2010
				Murakami et al. (2013)
	miR494-3p	TNF-α/PEDF	Candidate molecular targets for diagnosis and treatment	Mukai et al. (2021)
	miR-34	TP53/EGFR/JUN/BCL2	Inhibit tumor growth, metastasis, invasion and EMT	Zhang et al., 2007
				Nana-Sinkam and Croce, (2013)
	miR-184	mTOR	Affect AMD progress	Kuo et al., 2012
				Nomura et al., 2008
				Liu et al., 2014
				Jiang et al. (2016)
Osteoblasts	miR-449a	HDAC1/Runx2	Promote differentiation of iPS osteoblasts and growth stagnation of tumor cells	Buurman et al., 2012
				Jeon et al., 2012
				Okamoto et al. (2012)
	miR-211	Atg14	Promote cell differentiation	Ozeki et al. (2017)

IPSC-derived models are promising tools for deepening the understanding of early developmental processes (Dvash and Benvenisty, 2004). The major advantage of iPSC-derived models over primary cells is their capacity of repeatly generating cells with specific genetic background of the donors. With this property along with their pluripotency, hiPSCs can serve as a powerful tool for human cell replacement therapies and as an *in vitro* platform for personalized drug screening and discovery (Pouton and Haynes, 2007; Stadtfeld and Hochedlinger, 2010).

The reprogramming of somatic cells derived from patients and healthy donors into iPSCs is an important step to establish human-relevant models for illustrating the molecular and cellular mechanisms underlying the disease pathology. Notably, iPSCs can also be used to develop and test new therapies *in vitro*. Here, we discuss the regulatory role of miRNAs in iPSC-derived models for human development. In the future, miRNA-related studies need to be further improved to utilize hiPSCs as powerful tools in research of developmental biology. To address this issue, new methods, such as employing ectopic miRNAs as epigenetic modulators, should also be developed to optimize existing cell reprogramming and differentiation protocols (Ferreira et al., 2018).

There is a need to more thoroughly explore the role of miRNAs in human developoment. Given their relevance, we expect miRNAs to be exploited as diagnostic markers and as therapeutic targets for developmental diseases soon.
